# Homocysteine as a Risk Factor for Atherosclerosis: Is Its Conversion to *S*-Adenosyl-*L*-Homocysteine the Key to Deregulated Lipid Metabolism?

**DOI:** 10.1155/2011/702853

**Published:** 2011-08-01

**Authors:** Oksana Tehlivets

**Affiliations:** Institute of Molecular Biosciences, University of Graz, Humboldtstrasse 50/II, 8010 Graz, Austria

## Abstract

Homocysteine (Hcy) has been recognized for the past five decades as a risk factor for atherosclerosis. However, the role of Hcy in the pathological changes associated with atherosclerosis as well as the pathological mechanisms triggered by Hcy accumulation is poorly understood. Due to the reversal of the physiological direction of the reaction catalyzed by *S*-adenosyl-*L*-homocysteine hydrolase Hcy accumulation leads to the synthesis of *S*-adenosyl-*L*-homocysteine (AdoHcy). AdoHcy is a strong product inhibitor of *S*-adenosyl-*L*-methionine (AdoMet)-dependent methyltransferases, and to date more than 50 AdoMet-dependent methyltransferases that methylate a broad spectrum of cellular compounds including nucleic acids, proteins and lipids have been identified. Phospholipid methylation is the major consumer of AdoMet, both in mammals and in yeast. AdoHcy accumulation induced either by Hcy supplementation or due to *S*-adenosyl-*L*-homocysteine hydrolase deficiency results in inhibition of phospholipid methylation in yeast. Moreover, yeast cells accumulating AdoHcy also massively accumulate triacylglycerols (TAG). Similarly, Hcy supplementation was shown to lead to increased TAG and sterol synthesis as well as to the induction of the unfolded protein response (UPR) in mammalian cells. In this review a model of deregulation of lipid metabolism in response to accumulation of AdoHcy in Hcy-associated pathology is proposed.

## 1. Introduction

The first indication that sulfur amino acid metabolism is linked to atherosclerosis came from observations in 1953 demonstrating that pathogenic cholesterol concentrations and experimental atherogenesis in monkeys can be inhibited by dietary methionine [1]. Since the early 60s elevated Hcy levels in blood (hyperhomocysteinemia) caused by different deficiencies of sulfur amino acid metabolism were reported to be associated with vascular disease and, in particular, with atherosclerotic plaque formation [2, 3]. Today, Hcy is recognized by many studies as a strong, independent and causal risk factor for atherosclerosis [4–8], although there is still controversy on the underlying metabolic connections [9]. In addition to its association with vascular diseases, Hcy is also linked to neurological disorders [10], aging [11], and all-cause mortality [12]. Understanding the pathological mechanisms triggered by Hcy is, therefore, essential for understanding its role in several disease states. 

**Figure 1:**
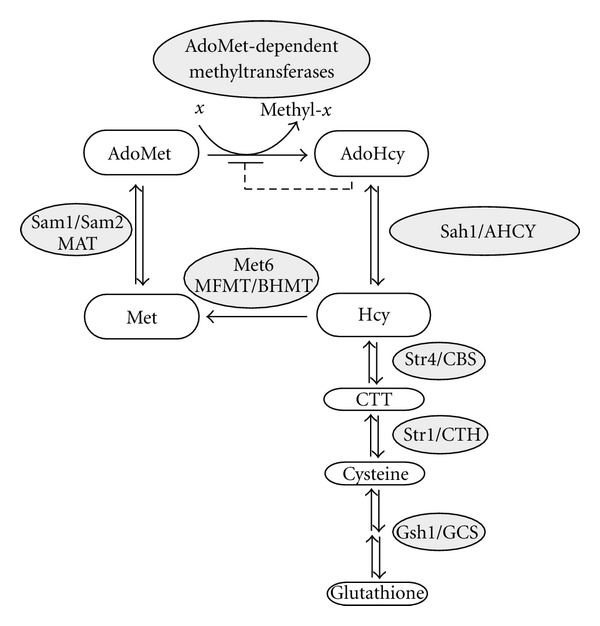
Role of AdoHcy and Hcy in AdoMet-dependent methylation in yeast and mammals. The enzymes involved in yeast and mammalian metabolism are shown in grey circles. AdoMet: * S*-adenosyl-*L*-methionine; AdoHcy:  *S*-adenosyl-*L*-homocysteine; Hcy: homocysteine; Met: methionine; CTT: cystathionine; in yeast: Sah1: *S*-adenosyl-*L*-homocysteine hydrolase; Sam1 and Sam2: *S*-adenosyl-*L*-methionine synthetases; Met6: methionine synthase; Str4: cystathionine *β*-synthase; Str1: cystathionine *γ*-lyase; Gsh1: *γ*-glutamylcysteine synthetase; in mammals: AHCY: *S*-adenosyl-*L*-homocysteine hydrolase; MAT: methionine adenosyltransferase; MFMT: 5-methyltetrahydrofolate homocysteine methyltransferase; BHMT: betaine homocysteine methyltransferase; CBS: cystathionine *β*-synthase; CTH: cystathionine *γ*-lyase; GSH: glutathione synthase.

Numerous mechanisms have been proposed that explain pathological changes associated with elevated Hcy levels (reviewed in [3]). Several of them, for example, protein homocysteinylation and oxidative stress, are directly triggered by Hcy. However, not Hcy, but rather AdoHcy, an immediate precursor of Hcy (Figure 1), emerged as a more sensitive indicator of cardiovascular disease during the last decade [13, 14]. Supporting the potentially pathogenic role of AdoHcy, studies in yeast showed that indeed AdoHcy is more toxic than Hcy to cells that are deficient in Hcy catabolism [15]. 

AdoHcy is synthesized as a universal byproduct of AdoMet-dependent methyltransferase reactions (Figure 1). It is a strong competitive inhibitor of many AdoMet-dependent methyltransferases [16] and, therefore, has to be removed to sustain these reactions. The only eukaryotic enzyme capable of AdoHcy catabolism, *S*-adenosyl-*L*-homocysteine hydrolase (Sah1 in yeast, AHCY in mammals), catalyzes the reversible hydrolysis of AdoHcy to Hcy and adenosine. The equilibrium of *S*-adenosyl-*L*-homocysteine hydrolase-catalyzed reaction lies far in the direction of synthesis, and both Hcy and adenosine have to be quickly metabolized in order to drive the net hydrolysis of AdoHcy [17]. Therefore, accumulation of hydrolytic products of the *S*-adenosyl-*L*-homocysteine hydrolase-catalyzed reaction, in particular Hcy, results in AdoHcy synthesis and accumulation showing that AdoHcy is not only the precursor, but also the product of Hcy metabolism* in vivo* [18–20]. 

Changes at the epigenetic level are the most extensively studied consequences of methylation deficiency [21–24]. However, phospholipid methylation that requires three sequential AdoMet-dependent methylation steps to synthesize one molecule of phosphatidylcholine (PC) from phosphatidylethanolamine (PE), the predominant way for PC synthesis in yeast, in particular, in the absence of choline and ethanolamine in the culture medium, is the major consumer of AdoMet. Phospholipid methylation is also the major consumer of AdoMet in mice, since the loss of phosphatidylethanolamine N-methyltransferase (PEMT) in PEMT^−/−^ knockout mice leads to a 50% decrease in plasma Hcy levels [25]. Reexamination of methylation metabolism in humans also revealed that phospholipid methylation, but not creatine synthesis, as was assumed previously, accounts for the major part of AdoMet being utilized in the human body [26].

While PE methylation is the predominant way to synthesize phospholipids in yeast, phospholipid synthesis by the *de novo* methylation pathway is primarily present in the liver in mammals, where it constitutes 30% of PC production and account for estimated 10 *μ*mol and 1,65 mmol PEMT-derived PC secreted into bile per day in mice and humans, respectively [26, 27]. However, other mammalian tissues and cells are also capable of phospholipid methylation including brain, skeletal muscle, adipose tissues, fibroblasts, arterial smooth muscle cells, endothelial cells, macrophages, and erythrocytes [28–37]. The evolutionary conservation of phospholipid methylation suggests its essential role in some specific functions in different cell types. For instance, phospholipid methylation is enhanced in hypertrophied myocardium, correlates with the level of *β*-adrenergic receptors [38, 39] and is stimulated by isoproterenol, a potent cardiac stimulant [40]. In contrast, phospholipid methylation is inhibited by quinidine, an antiarrhythmic drug that causes repression of myocardial contractility [41]. Phospholipid methylation was also observed in microsome preparations from aorta [42, 43] and was suggested to affect membrane fluidity and function of membrane calcium channels in aorta [42, 43] as well as in heart [40]. Moreover, phospholipid methylation appears to be coupled to Ca^2+^ influx and von Willebrand factor release in endothelial cells [35]. In accordance, it was shown that increased methylation of phospholipids is required for an influx of Ca^2+^ and subsequent release of histamine in mast cells [44]. Furthermore, Ca^2+^ influx was correlated with the release of arachidonic acid in rabbit neutrophils and human fibroblasts, which also appears to require phospholipid methylation [32, 45]. Requirement of phospholipid methylation for polyunsaturated fatty acid metabolism was also observed in the brain [46]. It was reported that developing, remyelinating, and diabetic brain exhibits increased synthesis of PC by the *de novo* methylation pathway in comparison with normal adult brain [47, 48]. Phospholipid methylation was shown to be linked to diabetes [49–51] and neurological disorders [52, 53] also in other studies.

PEMT mRNA and protein levels increase substantially in differentiating adipocytes [30]. It was shown very recently that phospholipid methylation is required for lipid droplet formation and stability in 3T3-L1 adipocytes, and high-fat challenge induces PEMT expression in adipose tissue [54]. Moreover, PEMT and the CDP-choline pathway for PC synthesis are both required for the secretion of very-low-density lipoproteins [55–57]. While cells lacking the rate-limiting enzyme of the CDP-choline pathway, CTP:phosphocholine cytidylyltransferase, do not survive [57], deficiency of phospholipid methylation in PEMT^−/−^ mice under choline deprivation results in development of hepatic steatosis followed by steatohepatitis and hyperacute liver failure [58] and is lethal within 5 days [59]. Moreover, deficiency in phospholipid methylation, but not in the synthesis of PC by the CDP-choline pathway, protects from diet-induced obesity in mice due to increased energy utilization suggesting that PEMT plays a role in whole energy metabolism and is linked to insulin signaling [60]. Finally, an isoform of phosphatidylethanolamine N-methyltransferase, PEMT2, appears to be involved in the control of hepatocyte cell division, since its inactivation is associated with several types of liver cell proliferation including tumorigenesis [61]. Vice versa, rat hepatoma cell growth is suppressed by PEMT2 expression [62].

Sensitivity of phospholipid methylation to AdoHcy accumulation [16, 18, 63] as well as numerous correlations reported for phospholipid methylation pathway suggests that interference with this reaction in Hcy-associated pathology may lead to widespread defects, what indeed seems to be the case. In particular, elevated Hcy levels were found to trigger deregulation of lipid metabolism in yeast and mammalian cells [18, 64]. A mechanism of deregulation of lipid metabolism and lipid-associated cellular functions in hyperhomocysteinemia mediated by AdoHcy accumulation and subsequent inhibition of phospholipid methylation is proposed in this paper. 

## 2. Role of Homocysteine in the Methylation Cycle

Homocysteine is a sulfur-containing amino acid, which does not occur in proteins, but is found at the intersection of methylation and transsulfuration metabolism (Figure 1, reviewed in [65]). Hcy is formed during methionine metabolism by *S*-adenosyl-*L*-homocysteine hydrolase that catalyzes the reversible hydrolysis of AdoHcy to Hcy and adenosine. To be kept in the methylation cycle, Hcy has to be remethylated to methionine, which can be further activated to AdoMet and used by over 50 AdoMet-dependent methyltransferases that release AdoHcy as a by-product after the methyl transfer reaction. The ratio of AdoMet to AdoHcy, that is, the ratio of the substrate versus the specific inhibitor of AdoMet-dependent methyltransferases, is indicative of the cellular methylation potential [66].

In addition to its remethylation to methionine, Hcy can be subjected to transsulfuration leading to the synthesis of cysteine, which is also a precursor of glutathione, an essential cellular defense molecule in oxidative stress response [65]. This pathway irreversibly withdraws Hcy from the methylation cycle. An alternative way for Hcy metabolism is the reversal of the reaction catalyzed by *S*-adenosyl-*L*-homocysteine hydrolase. This occurs upon accumulation of the hydrolytic products of the reaction, in particular Hcy, and leads to AdoHcy synthesis and accumulation [19, 67–69]. Thus, elevated Hcy levels via accumulation of AdoHcy lead to the disruption of the methylation cycle and, potentially, to methylation deficiency.

Deficiency in cystathionine *β*-synthase (CBS), the first and rate-limiting enzyme of the transsulfuration pathway (Figure 1), is the major cause of severe hyperhomocysteinemia followed by genetic defects of folate and cobalamin metabolism that is involved in Hcy remethylation [65]. These pathological conditions lead to the plasma Hcy levels of more than 100 *μ*mol/L [65] and are rare in comparison with mild hyperhomocysteinemia that is caused by dietary deficiencies of the vitamin cofactors required for Hcy catabolism - folic acid, vitamins B_6_ and B_12_, and characterized by the plasma Hcy levels of 15–25 *μ*mol/L [70]. Vitamin B_6_ is required for the activity of CBS. Folic acid and vitamin B_6_ are required for the activity of methionine synthase catalyzing 5-methyltetrahydrofolate-dependent remethylation of Hcy to methionine (Figure 1). While vitamin supplementation appeared to be a straightforward strategy to reduce/prevent cardiovascular events, this possibility was studied in several large trials. However, it was observed that vitamins, while capable of lowering elevated plasma Hcy levels, do not reduce the rates of vascular events [71]. Several potential mechanisms that might explain this result by offsetting the positive effect of Hcy-lowering therapy were subsequently proposed. These include promotion of cell proliferation by folic acid through its role in the synthesis of thymidine, increase of the methylation potential leading to changes in gene expression, and increase in the levels of asymmetric dimethylarginine that inhibit the activity of nitric oxide synthase [71]. An additional possibility is that, not Hcy, but rather a related metabolite could be a trigger of some pathological changes associated with elevated Hcy levels. Possibly explaining the failure of Hcy-lowering vitamins to reduce vascular events, it was recently reported that supplementation with B-vitamins including folate does not efficiently lower plasma AdoHcy levels [72], presumably due to elevation of AdoMet-dependent methylation. 

## 3. AdoHcy-Triggered Deregulation of Lipid Metabolism in Yeast

In yeast, the synthesis of PC from PE by the *de novo* phospholipid methylation pathway is particularly sensitive to AdoHcy accumulation [18, 63]. Both inhibition of *S*-adenosyl-*L*-homocysteine hydrolase and Hcy supplementation results in AdoHcy accumulation and inhibition of phospholipid methylation in yeast [18]. However, not only phospholipid methylation, but also a methylation-independent branch of lipid metabolism, namely, TAG synthesis, is affected by AdoHcy accumulation in yeast: yeast cells deficient in AdoHcy catabolism or supplemented with Hcy massively accumulate TAG [18]. Supporting the causal role of impaired phospholipid methylation in the deregulation of TAG metabolism in response to AdoHcy accumulation, it was found that yeast mutants that are deficient in the enzymatic activities required for methylation of PE to PC, *cho2* and *opi3,* also accumulate TAG [18]. TAG is known to play an important role in buffering excess fatty acids [73]. Therefore, TAG accumulation under these conditions suggests accumulation of fatty acids and their redirection from phospholipid to TAG synthesis in methylation deficiency in yeast.

Another observation as well supports accumulation of fatty acids under these conditions. In addition to TAG metabolism, transcriptional regulation of phospholipid biosynthesis is also affected in yeast mutants deficient in AdoHcy catabolism. Impaired phospholipid methylation in Sah1-depleted cells unable to hydrolyze AdoHcy or in *cho2* and *opi3 *mutants leads to upregulation of genes, which have an inositol-sensitive upstream regulatory sequence (UAS_INO_) in their promoter regions, indicating accumulation of the phospholipid precursor, phosphatidic acid, in the ER [18]. *ACC1* encoding acetyl-CoA carboxylase, the first and rate-limiting enzyme of fatty acid biosynthesis, is also a subject to UAS_INO_-mediated regulation, suggesting upregulation of the *de novo* fatty acid biosynthesis in response to AdoHcy accumulation. Moreover, Sah1 depletion also affects sterol synthesis in yeast, leading to 4-fold elevated squalene levels and suggesting accumulation of early precursors of ergosterol biosynthesis under these conditions (Tehlivets, Kohlwein, unpublished). Taken together, inhibition of phospholipid methylation induced by AdoHcy accumulation appears to lead to upregulation of fatty acid, TAG, and sterol biosynthetic pathways in yeast. 

## 4. Phospholipid Methylation and Homocysteine: Impact on Lipid Metabolism in Mammals

AdoHcy inhibits phosphatidylethanolamine N-methyltransferase *in vitro* and *in vivo* also in mammals [28, 37, 74]. Similarly as in yeast, deficiency of phospholipid methylation in PEMT^−/−^ knockout mice leads to a rapid decrease of the hepatic PC/PE ratio and accumulation of TAG in the liver, in the absence of choline supplementation [75]. However, TAG accumulation in the livers of these animals appears to be at least in part due to decreased TAG secretion from hepatocytes [55]. 

Elevated levels of Hcy are as well linked to deregulation of lipid metabolism in mammals. CBS^−/−^ knockout mice exhibit severe hyperhomocysteinemia and accumulate AdoHcy in all tissues tested [68, 69]. These mutant animals show elevated TAG and nonesterified fatty acid levels in the liver and serum and develop hepatic steatosis [76, 77]. Another genetic disorder that results in moderately elevated Hcy levels, methylenetetrahydrofolate reductase (MTHFR) deficiency, leads to fatty liver development as well as to neuropathology and aortic lipid deposition in mouse models [78, 79]. Dietary-induced hyperhomocysteinemia in mice also causes fatty liver, further supporting the role of Hcy in deregulation of lipid metabolism in mammals [64]. In these mice as well as in the CBS^−/−^ knockout mice lipid accumulates in liver rather than in serum [64, 76]. 

Preferable accumulation of lipids in the liver and, possibly, other tissues in hyperhomocysteinemia suggests that other mechanisms than those associated with elevation of circulating lipids are responsible for the development of cardiovascular disease under these conditions. Indeed, conventional risk factors including hypercholesterolemia accounts only for approximately 50% of all cases of cardiovascular disease, while 40% of patiens diagnosed with premature coronary artery disease, peripheral vascular disease or venous thrombosis exhibit hyperhomocysteinemia [80]. In accordance, unlike typical lipid-rich atherosclerotic plagues, vascular lesions associated with hyperhomocysteinemia are lipid-poor, fibrous plaques [81, 82], greatly outnumbering fatty atherosclerotic lesions [80]. 

In animal models of hyperhomocysteinemia atherosclerotic lesions are rare. They are found only in the MTHFR^−/−^ knockout mice that exhibit aortic lipid accumulation reminiscent of early atherosclerotic lesions [2, 78, 83], however, not in, for example, CBS^−/−^ knockout mice. This discrepancy might be due to disruption of two different Hcy utilizing pathways in these animals. While 5-methyltetrahydrofolate-dependent Hcy remethylation occurs in all mammalian cells, transsulfuration of Hcy occurs primarily in the liver and kidney [65]. Thus, impairment of 5-methyltetrahydrofolate-dependent Hcy remethylation in MTHFR^−/−^ knockout mice may differently affect Hcy metabolism in comparison to the deficiency in the first step of Hcy transsulfuration in CBS^−/−^ knockout mice. The observation that dietary (methionine or Hcy supplementation) or genetically (CBS gene deletion) induced hyperhomocysteinemia in apoE-deficient (apoE^−/−^) mice leads to development of larger and more advanced atherosclerotic lesions clearly demonstrates a causal relationship between elevated Hcy levels and atherosclerosis [83]. In contrast, lack of PEMT was shown to reduce significantly plasma VLDL and to attenuate atherosclerosis in both PEMT^−/−^/Ldlr^−/−^ mice deficient in PEMT and LDL receptors as well as in PEMT^−/−^/ApoE^−/−^ mice [84, 85]. 

## 5. Role of the Unfolded Protein Response in Hyperhomocysteinemia and Atherosclerosis

Elevated Hcy levels induce endoplasmic reticulum (ER) stress and activate the unfolded protein response (UPR) in a variety of mammalian cells. These include cultured human hepatocytes, vascular endothelial and aortic smooth muscle cells as well as liver cells of the CBS^−/−^ knockout mice [64, 86–88]. Furthermore, elevated Hcy levels lead to activation of the sterol regulatory element-binding proteins (SREBPs), which function to activate genes encoding enzymes in cholesterol, fatty acid, and TAG metabolism and uptake, both in cultured mammalian cell lines as well as in the livers of the CBS^−/−^ knockout mice [64, 86]. ER stress appears to play a direct role in the activation of TAG and cholesterol biosynthesis, since overexpression of the ER chaperone GRP78/BiP was reported to inhibit Hcy-induced SREBP-1 gene expression in mammalian cell cultures [64] as well as in mice [89] and lead to reduction of the hepatic steatosis in leptin-deficient (*ob/ob*) mice [89]. SREBP-1 overcomes translation inhibition induced by UPR through an internal ribosome entry site (IRS), similarly to GRP78 [90]. 

Confirming the causal role of Hcy in UPR induction and deregulation of lipid metabolism, a decrease of elevated plasma Hcy levels is accompanied by a decrease in hepatic lipids and ER stress response [91]. A strong correlation between lipid metabolism, ER stress response and elevated Hcy levels is also evident form a literature mining approach [92]. Further demonstration of close relationship between ER stress and Hcy metabolism came from the observation that MTHFR involved in Hcy remethylation is induced in response to ER stress [93]. Evolutionary conservation of the relationship between Hcy and UPR is shown by the induction of ER stress and activation of UPR in response to Hcy supplementation in yeast [94]. Finally, demonstrating its pathophysiological role, ER stress was shown to be strongly associated with accelerated atherosclerosis in hyperhomocysteinemic apoE-deficient mice [95], liver diseases [96] as well as hyperglycemia-induced atherosclerosis [97]. 

## 6. Deregulation of Fatty Acid Metabolism in Response to AdoHcy Accumulation

The UPR, as a conserved cellular stress response pathway, is aimed at restoring normal ER and secretory function as well as membrane trafficking upon impaired protein folding in the ER. The *de novo* methylation and the CDP-choline phospholipid biosynthetic pathways produce phospholipid species with distinct fatty acyl chain composition in yeast: the *de novo* phospholipid methylation pathway produces more unsaturated phospholipids [98, 99]. Similarly, the PEMT-generated PC pool in mammals is also enriched in unsaturated fatty acids [100, 101]. Supporting the role of PEMT in metabolism of unsaturated fatty acids PEM^−/−^ knockout mice were reported to accumulate more saturated PC molecular species in the liver compared with the control littermates [60] and to exhibit dramatically reduced concentrations of polyunsaturated fatty acids in the plasma and in hepatic PC, independently of choline status [102]. Thus, beyond its role as a compensatory pathway for PC biosynthesis under conditions of choline deprivation, phospholipid methylation plays a crucial role in unsaturated fatty acid metabolism both in yeast and in mammals. The observation that deficiency of phospholipid methylation in *cho2* and *opi3* yeast mutants is synthetically lethal in the absence of a functional UPR [103] suggests an essential requirement of UPR in response to impaired phospholipid methylation. Thus, Hcy accumulation, which was shown to lead to AdoHcy-mediated inhibition of phospholipid methylation in yeast, may lead to accumulation of saturated fatty acids in membrane phospholipids—a potential pathological mechanism that might be shared by both yeast and mammals.

Indeed, accumulation of saturated fatty acids in membrane phospholipids interferes with ER structure and integrity, induces ER stress and leads to cell death in mammalian cell cultures [104, 105]. Accordingly, decreased membrane phospholipid desaturation due to stearoyl-CoA desaturase 1 knockdown induces UPR in HeLa cells [106]. Vice versa, overexpression of stearoyl-CoA desaturase attenuates palmitate-induced ER stress and protects from lipoapoptosis [107–109]. Treatment with the molecular chaperone 4-phenyl butyrate, which is capable of stabilization of protein conformation, improvement of ER folding capacity and facilitation of protein trafficking, leads to abolishment of UPR induction in yeast subjected to lipid-induced ER stress [105]. This finding suggests that accumulation of saturated fatty acids in membrane phospholipids first leads to changes in the membrane environment followed by induction of ER stress and accumulation of misfolded protein(s) that, in turn, activate UPR [105]. Potential mechanisms involved in saturated fatty acid-induced UPR include depletion of ER Ca^2+^ stores leading to decreased ER chaperone activity and protein misfolding, as well as interference with ER-to-Golgi trafficking [110]. Recently, proteomic studies showed that carboxypeptidase E, a key enzyme involved in processing [111] and sorting of insulin [112], is involved in induction of ER stress in *β*-cells in response to palmitate treatment [113]. Degradation of carboxypeptidase E in palmitate-induced ER stress is mediated by palmitate metabolism and Ca^2+^ flux [113]. Alternatively, changes of the ER membrane environment may directly activate ER sensors IRE1, ATF6, and PERK or modulate the binding of the ER sensors to the ER chaperone GRP78 causing its dissociation and activation of UPR pathways. 

Supporting the hypothesis that Hcy interferes with phospholipid acyl chain composition it was observed in humans that elevated plasma AdoHcy levels are negatively correlated with both PC content and the level of polyunsaturated fatty acids in PC, but not in PE, in red blood cells in Alzheimer's patients [114]. Elevated plasma Hcy levels were also shown to be associated with a decrease in polyunsaturated (docosahexaenoic) fatty acids in the plasma of healthy humans [115] and in the plasma and erythrocytes of cystic fibrosis patients; these individuals exhibit increased Hcy and AdoHcy levels as well as altered PE and PC metabolism [116].

**Figure 2:**
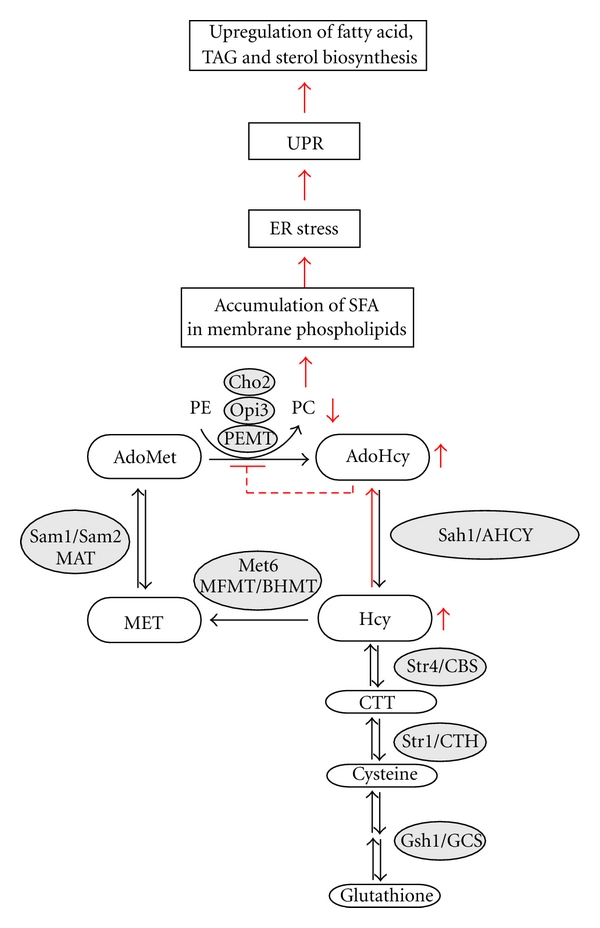
A model of activation of UPR and upregulation of fatty acid, TAG, and sterol biosynthesis in response to inhibition of phospholipid methylation in hyperhomocysteinemia. Elevated Hcy levels via AdoHcy accumulation and inhibition of phospholipid methylation lead to accumulation of saturated PC molecular species in ER membranes followed by ER stress, UPR activation, and upregulation of fatty acid, TAG, and sterol biosynthesis. The enzymes involved in yeast and mammalian metabolism are shown in grey circles (see Figure 1). PE: phosphatidylethanolamine; PC: phosphatidylcholine; PEMT: phosphatidylethanolamine N-methyltransferase in mammals; *Cho2* and *Opi3*: phosphatidylethanolamine N-methyltransferases in yeast.

Taken together, deficiency of phospholipid methylation caused by AdoHcy accumulation in Hcy-associated pathology appears to lead to an increase in saturated PC molecular species in ER membranes followed by ER stress, protein misfolding, induction of UPR, and activation of lipid metabolism (Figure 2). Upregulation of lipid biosynthesis, which apparently should serve to compensate for suboptimal composition of membrane lipids, leads, however, to accumulation of fatty acids, TAG and sterols in the absence of functional phospholipid methylation. In addition to a role in formation of a specific pool of PC molecular species, phospholipid methylation is crucial for maintenance of a distinct PC/PE ratio important for cell integrity when dietary choline-supply is blunted. Decrease of the PC/PE ratio was reported to result in increased cell permeability of hepatocytes from PEMT^−/−^ mice fed choline deficient diet leading to liver damage [75]. Similarly, decrease of PC or increase in PE was shown to lead to cell damage and/or death in several other mammalian cell types [117–119]. Finally, observation of different outcomes in hyperhomocysteinemic apoE^−/−^ mice and both in PEMT^−/−^/Ldlr^−/−^ and PEMT^−/−^/ApoE^−/−^ mice suggests mechanisms besides inhibition of phospholipid methylation, for example, AdoHcy-dependent modulation of gene expression that may also contribute to the development of Hcy-dependent atherosclerosis. 

## 7. Concluding Remarks

The metabolism of homocysteine, consequences of its accumulation as well as associated AdoHcy-triggered inhibition of AdoMet-dependent methylation are complex. In this paper a novel mechanism of Hcy-triggered deregulation of lipid metabolism and UPR induction that is mediated through an AdoHcy-dependent inhibition of phospholipid methylation and based on experimental evidence derived from both yeast and mammalian systems is proposed. The observations made in yeast and mammals are summarized in Table 1. 

*S*-adenosyl-*L*-homocysteine hydrolase is recognized since many years as a target for antiviral drug design [120]. Inhibitors that block AdoHcy hydrolysis are efficient against many types of viruses including Ebola and show other effects of pharmacological importance [120–122]. However, they are associated with high cytotoxicity due to interference with central metabolic pathways [121, 122]. While using these inhibitors to study the effects of AdoHcy accumulation appears to be straightforward, ability of some of nucleoside inhibitors of *S*-adenosyl-*L*-homocysteine hydrolase to undergo metabolic phosphorylation to nucleotides may account for a part of their biological activities [121, 122]. Inhibitors that would be able to block selectively homocysteine and adenosine conversion to AdoHcy are not available. However, based on current understanding of regulation of homocysteine and methionine metabolism (i) selective blockage of AdoHcy synthesis from homocysteine and adenosine that will relieve not only inhibition of phospholipid methylation but also many other AdoMet-dependent methyltransferase reactions, combined with (ii) vitamin B_6_ supplementation in order to accelerate homocystein catabolism by transsulfuration pathway, may serve as a way to reduce AdoHcy in hyperhomocysteinemia without elevation of AdoMet.

Many questions in the model presented in this paper are still unanswered. Is AdoHcy-mediated accumulation of saturated fatty acids in membrane lipids indeed the way by which elevated Hcy levels induce ER stress? What role do specific lipid precursors have in regulation of lipid metabolism in UPR? What is the impact of AdoHcy accumulation on other methylation reactions unrelated to lipid metabolism? What is the role of deficient phospholipid methylation in homocysteine-associated pathology beyond deregulation of fatty acid, TAG, sterol metabolism, and UPR induction? Elucidation of the molecular mechanisms triggered by elevated Hcy levels will undoubtfully improve our understanding of its pathological role in numerous diseases. 

## Figures and Tables

**Table 1:** Experimental evidence on deregulation of lipid metabolism and UPR induction under elevated homocysteine levels in yeast and mammals*.

*Experimental evidence*	*Yeast*	*Mammals*
AdoHcy is formed *in vivo* in response to elevated Hcy levels	+	+

AdoHcy is more toxic than Hcy to cells deficient in Hcy catabolism	+	

AdoHcy represents a better marker of cardiovascular risk than Hcy		+

Phospholipid methylation is quantitatively the major consumer of AdoMet	+	+

Phospholipid methylation is inhibited in response to Hcy supplementation	+	

Phospholipid methylation is inhibited by AdoHcy	+	+

TAG is accumulating in response to Hcy supplementation	+	+

TAG is accumulating in response to deficiency in AdoHcy hydrolysis	+	

TAG is accumulating in response to deficiency in phospholipid methylation	+	+

UPR is inducted in response to Hcy supplementation	+	+

The *de novo* phospholipid methylation pathway produces phospholipids enriched in unsaturated fatty acids	+	+

ER stress is inducted by accumulation of saturated fatty acids in membrane phospholipids	+	+

Hcy/AdoHcy levels are inversely correlated to the levels of unsaturated fatty acids		+

*The absence of a plus sign in some columns implies lack of data or nonapplicability.

## Abbreviations

AdoMet: *S*-adenosyl-*L*-methionine
AdoHcy: *S*-adenosyl-*L*-homocysteine
Hcy: Homocysteine
PC: Phosphatidylcholine
PE: Phosphatidylethanolamine
TAG: Triacylglycerol
UPR: Unfolded protein response
Sah1: *S*-adenosyl-*L*-homocysteine hydrolase in yeast
AHCY: *S*-adenosyl-*L*-homocysteine hydrolase in mammals
Cho2 and Opi3: Phosphatidylethanolamine N-methyltransferases in yeast
PEMT: Phosphatidylethanolamine N-methyltransferase in mammals
CBS: Cystathionine *β*-synthase
MTHFR: Methylenetetrahydrofolate reductase.
